# Unlocking the secrets of exchange rate determination in Malaysia: A Game-Changing hybrid model

**DOI:** 10.1016/j.heliyon.2023.e19140

**Published:** 2023-08-14

**Authors:** Shamaila Butt, Muhammad Ramzan, Wing-Keung Wong, Muhammad Ali Chohan, Suresh Ramakrishnan

**Affiliations:** a Accounting and Finance Department, Faculty of Management, University Technology Malaysia, Block T08, 81310 UTM Skudai, Johor, Malaysia; b Faculty of Management and Administrative Sciences, Department of Business Administration, University of Sialkot, Punjab, Pakistan; c Department of Finance, Fintech & Blockchain Research Center, And Big Data Research Center, Asia University 500, Lioufeng Road, Wufeng, Taichung, Taiwan, 41354; d Department of Medical Research, China Medical University Hospital No.91, Hsueh-Shih Road, Taichung, Taiwan Postal Code: 40402, R.O.C. Department of Economics and Finance, The Hang Seng University of Hong Kong, Hang Shin Link, Siu Lek Yuen, Shatin, New Territories, 999077, Hong Kong; e Faculty of Business. Sohar University, 3111 Al Jamiah Street, Sohar, 311 Oman

**Keywords:** Microstructure approach, Oil price, Order flow, Bid-ask spread, Nonlinear ARDL

## Abstract

Nominal exchange rate determination is a puzzling phenomenon throughout the literature. Thus, the study aims to analyze the nominal exchange rate determination with a hybrid approach of macroeconomic and microstructure determinants, i.e., interest rate differential, oil price, order flow, and bid-ask spread over the long- and short-run horizons in the context of Malaysia. The dataset consists of high-frequency daily data from 2010 to 2017, employing a nonlinear ARDL approach. The results indicate that the bid-ask spread and interest rate differential were found to be key determinants of exchange rate dynamics in the long and short run. The findings show strong evidence of long-run asymmetry in the interest rate differential, while short-run asymmetry effects exist between microstructure determinants and the exchange rate. In addition, it indicates that the bid-ask spread holds informative content to explain the dynamics of the exchange rate in Malaysia. Additionally, the negative changes in the oil price could potentially act as macroeconomic news announcements and the bid-ask spread as liquidity determinants in Malaysia, which play a significant role in exchange rate determination. The study concluded that the prominent short-run asymmetry effects captured in cumulative order flow and bid-ask spread While a long-run asymmetry exists between the oil price and exchange rate in Malaysia. The empirical results allow for long-run and short-run asymmetric pricing impacts of a hybrid approach on the nominal exchange rate in Malaysia. This study is helpful in providing policy direction and practical implications for monetary authorities and market dealers. The bid-ask spread and oil price could be considered influential exchange rate determinants in the short run in Malaysia.

## 1 Introduction

The exchange rate market is the biggest trading market by trading volume all over the globe [1]. The foreign exchange market, or forex market, has received considerable attention across the globe from academicians and industry because of its dynamic and unprecedented nature [2,3]. Particularly, exchange rates have drawn substantial attention in emerging economies as they are one of the key indicators of unpredictability and uncertainty [4,5]. The exchange rate determination is significantly influential on the various aspects of the economy and the stakeholders [6]. The role of the exchange rate is crucial in the global market, and its variability is proportional to the economic performance of a country [7,8].

Despite a reasonable contribution, several studies highlighted the shortcomings of macroeconomic fundamentals in the determination of exchange rates. Past studies by Refs. [9,10] argued that macroeconomic models contain limited information for determining exchange rates. While the microstructure approach offered a different perspective for understanding exchange rate movements [11]. The microstructure approach emphasizes that certain information is inaccessible to the public but is disseminated among traders [12]. By gradually combining public and non-public information in a hybrid approach, the trading process plays a crucial role in exchange rate determination. The hybrid approach performs better than the macro approach [[13], [14], [15]].

Besides the microstructure approach, the oil price changes potentially act as observable macroeconomic news to explain the daily movement of exchange rate in commodity currency economies [16]. highlighted that oil price is an important factor for exchange rate determination as macroeconomic news announcements. They demonstrated that at high frequencies, exchange rate is influenced by microstructure approach, with order flow emerging as the most significant predictor. The order flow is a non-economic determinant, thus to combine with oil price as economic determinant (macro-news), enables to provide greater explanatory power of exchange rate. Hence, this study emerged a new approach which combines the microstructure determinants, such as order flow and bid-ask spread, and oil price to explain the exchange rate dynamics at high frequency. To the best of researchers’ knowledge, there is limited evidences to show the oil price role as macro news on nominal exchange rate in emerging economies at high frequency data. Thus, this study emerged the concept of microstructure determinants and oil price in body of knowledge to examine the nonlinear behavior of exchange rate in long-run and short-run in Malaysia. Therefore, this study aims to determine the nonlinear relationship between exchange rate and microstructure determinants and oil price in long-run and short-run in Malaysia.

The remainder of the article is organized as follows: Section 2 explains the literature review, and Section 3 introduces the models and data, followed by the results and discussion in Section 4. Section 5 summarizes the paper.

## 2 Literature review

The market microstructure concept mainly discussed two theoretical models for exchange rate determination, such as the Kyle auction model introduced by Ref. [17] and the portfolio-shift model introduced by Ref. [18]. The Kyle auction model is an intuitive microstructure workhorse model that embodies the general logic of the microstructure approach. In this model, there are three players at equilibrium: sellers, buyers, and the auctioneer [18]. propose theoretical microstructure models for the foreign exchange market, termed hot-potato trading and portfolio-shift models. The two models frame actual market behaviors in the foreign exchange market. In particular, the portfolio-shift model is formulated based on the actual foreign exchange trading process, which explains how the key determinant of order flow would impact exchange rates at high frequency. The portfolio-shift model explicitly describes the connection between exchange rates and order flow and can be adopted directly to do the econometric estimation. Hence [15], elaborates on the exchange rate mapping mechanism of the hybrid approach of macro-micro fundamentals to analyze the movement in exchange rate dynamics. Furthermore, the microstructure approach focuses on examining the information transmission towards exchange rates in the foreign exchange market impounded into the spot exchange rate through the trading process. The foreign exchange trade is an integrated part of price formation, through which the spot exchange rate is determined and evolved. In contrast, macroeconomic exchange rate models ignore trading behavior. In addition, the details of trade orders, such as quotes, prices, and trade places, are not important over months, quarters, or longer.

Order flow is the crucial determinant in the transmission of relevant information to the exchange rate. The positive (negative) order flow signals buying (selling) pressure, suggesting positive (negative) returns. One of the most prominent studies by Ref. [18] documented that the exchange rate appreciation is due to an increase in order flow volume and positively traded transactions. Extensive empirical studies have supported the positive linkage between order flow and exchange rates in the shorter period [[19], [20], [21], [22]]. In contrast, past studies lack considerable evidence of the long-run cointegrating association between order flow and exchange rate [[23], [24], [25]]. The unresolved cointegrating relationship arguments are caused by the nonlinear and asymmetric behavior of the exchange rate market toward news announcements [26,27]. In this regard, the asymmetric price discovery process in the forex market was not disclosed in the existing microstructure literature.

Besides, the relationship between exchange rate and order flow with trade-size clustering indicates the limitation of price discovery [28,29]. Due to price discovery limitations, the order flow is not being observed for illiquid currencies. It shows that the order flow of liquid currency tends to be greater than that of illiquid currency [30]. confirms that traders are incapable of adjusting spot prices in the order flow to take into account the newly acquired fundamental information and the possibility of liquidating the market without much trading activity. Similarly [31], supported the argument of the less explanatory power of order flow for infrequent trading currencies. Hence, it infers that the order flow does not determine the exchange rate for less traded or illiquid currencies.

Moreover, liquidity is another prominent defining factor of the microstructure approach to determining the exchange rate [32]. In the forex market, liquidity commonly determines the different trade activities, such as trade density, bid-ask spread (spread), and transaction cost [33]. Liquidity is used to match the buyers' and sellers' currency rates. Past studies explain the significant role of the bid-ask spread on the nominal exchange rate. The bid-ask spread has a significant impact on the exchange rate [[34], [35], [36]]. Similar findings were also observed by Ref. [31]. They found that the bid-ask spread positively influenced the exchange rate in low-traded currencies [2]. also documented the positive link between bid-ask spread and exchange rate in the Ugandan economy for a longer and shorter period.

Further, oil price fluctuations plausibly act as a prominent macroeconomic news indicator to determine the exchange rate in commodity currency economies [37]. Besides [38,39], suggested that oil prices are unresponsive to the macroeconomic news announcement, indicating that they immediately reflect the other asset price information. Similar results were also reported by Ref. [16], who found that oil as “news” is a determinative factor of the exchange rate in high-frequency data. The explanatory power of the oil price with other determinants plays a major part in the exchange rate movement at high frequencies [40]. [16,41] argued for a noticeable linkage between exchange rate and order flow in high-frequency data as a non-economic determinant. Considering the fact that order flow combined with the oil price as an economic determinant (macro-news) enables greater explanatory power of the exchange rate, this concept confirms the findings of the study of [14], who reported that the hybrid approach of macro-micro determinants conveys high information transmission in a short period of time for exchange rate determination.

The novel studies documented the oil price and exchange rate connection using high-frequency data [40]. discussed that oil price acts as a piece of macroeconomic news explanatory factor, and [16] found a moderate correlation between the exchange rate and oil price in the shorter period. Though there is a considerably different influence of oil-exporting and importing economies on the exchange rate movement [[42], [43], [44]]. An increasing trend in oil prices caused an appreciation of domestic currency for the oil-exporting economy [45]. In contrast, an oil-importing economy will increase trade deficits that cause a debt burden and eventually depreciate the currency. Similar results were found by Refs. [46,47] in investigating the relationship between the variables. Furthermore, the past study of [48] explains the positive and significant relationship between productivity differentials, oil prices, gross capital formation, and RER appreciation. These studies postulated that an upsurge in oil prices leads to wealth transfers from an oil-importing country to an oil-exporting country, which improves trade deficits and results in the appreciation of the domestic currency.

Among macroeconomic fundamentals, the interest rate differential is the main factor influencing the foreign exchange market in the short term [49,50]. argue that interest rate differential contains information on impeding changes in the exchange rate movement that affect the forex market at high-frequency data. In a similar way [21,22,51], confirm that the rising interest rate differential led to the depreciation of the domestic currency because of lower demand. By contrast, a prominent study by Ref. [14] demonstrated that an immediate dollar appreciation was caused by an increase in interest rates. Several studies found a negative association between variables, supported by Refs. [52,53]. The studies documented that a rise in the interest rate differential would attract foreign investment, resulting in the appreciation of the domestic currency [54]. claimed the influence of interest rate differentials on the US Dollar and Chinese RMB, which allow dollar depreciation. Meanwhile [2], show negative but insignificant results consistent with [55]. Furthermore [48], documented that various empirical studies have examined the pass-through effect of the exchange rate.

Consequently, order flow is a predominant indicator of highly traded currencies. While the bid-ask spread is an influential contributing factor to explaining the daily movement of the exchange rates on illiquid or less traded currencies, Thus, there is a need to indicate the bid-ask spread as the dominant determinant of the foreign exchange market in emerging economies or low-traded currencies. However, limited attention has been paid to showing the link between macroeconomic (interest rate differential) and microstructure determinants (order flow and bid-ask spread) in emerging markets. Therefore, to fill this literature gap, the current research paper motivates the evaluation of the existence of a significant nonlinear relationship between the nominal exchange rate and macro-micro determinants (order flow, bid-ask spread, and interest rate differential) in Malaysia as an emerging economy.

Along with this, extensive research tends to focus on the relationship between oil prices and exchange rates in developed economies [16,40]. However, less consideration has been given to examining the oil price as a macro-news indicator with micro-determinants to improve exchange rate determination in emerging economies. Besides, order flow and oil prices contain information asymmetry in the forex market, which is beneficial to controlling liquidity risk in economies. This is the novelty of this paper: adding the oil price with microstructure determinants to give more details on the asymmetry of information transmission to the exchange rate. By addressing this gap, the current research study aims to explain the nonlinear relationship between the Malaysian exchange rate and a hybrid approach of microstructure determinants and oil prices over a longer and shorter period.

## 3 Data and methodology

**Fig. 1:**
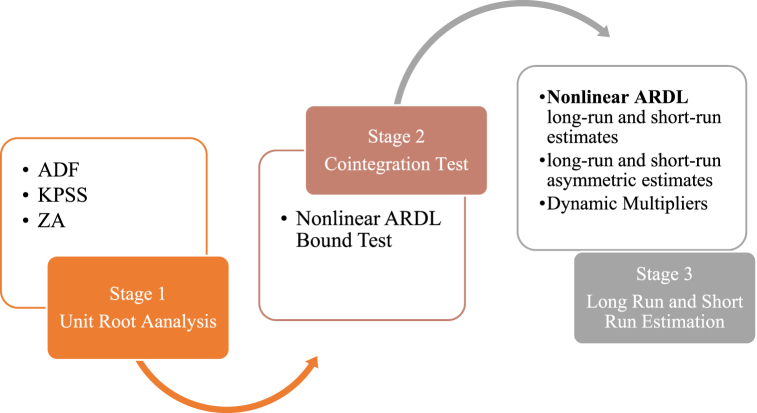
Steps of econometric modelling strategy.

This study used the daily dataset from January 4, 2010 to December 29, 2017, covering 1996 observations excluding weekends and public holidays. The bilateral exchange rate is measured as the Malaysian Ringgit per US Dollar. Order flow data is calculated by the tick test method, wherein the buy trade is counted as +1 and the sell trade is counted as −1. Further, the spread is the deviation between the ask and bid prices. The short-term interest rate differential is calculated by taking the difference between domestic and foreign overnight interest rates. The data are collected from different sources, such as Bank Negara Malaysia [56], Federal Reserve Economic Data (FRED), the Organization of the Petroleum Exporting Countries [57], Department of Statistics [58], and Bloomberg. Table 1 presents the sources and description of the data, and the data is stated in a natural logarithm with the exception of interest rate and order flow. Fig. 1 outlines the econometric methodology considered in the study.

**Table 1:** Sources and description of data.

Variables	Description	Data Sources
Nominal exchange rate (NER)	The price of a US Dollar in terms of Malaysian Ringgit	Bank Negara Malaysia
Interest rate differential (IRSD)	The Malaysian overnight policy rate minus the Federal overnight rate	Department of Statistics Malaysia and Federal Reserve Economic Data
Cumulative Order Flow (OF)	Buyer-initiated minus seller-initiated (1-min tick-by-tick orders)	Bloomberg
Bid-Ask Spread (BAS)	Ratio of ask and bid price of the currency rate	Bank Negara Malaysia
Oil price (OP)	OPEC (Basket price per barrel)	Organization of the Petroleum Exporting Countries

### 3.1 Theoretical consideration

Unlike traditional macroeconomic models, which rely exclusively on public information [14], proposed a portfolio shift model or hybrid model for the determination of the exchange rate. The hybrid model incorporates both macroeconomic and microstructure information to resolve the challenges posed by macroeconomic models. Thus, the study aims to formulate the exchange rate determination within the portfolio shift model as stated in Eq. (1):

(1) $${\Delta lnNER}_{t}=\Delta X_{t}+\Delta W_{t}+\Delta I_{t}$$

where $\Delta {lnNER}_{t}$ denotes the changes in the spot exchange rate, $\Delta X_{t}$ is daily cumulated net order flows, $\Delta W_{t}$ represents other microstructure determinants, and $\Delta I_{t}$ shows macroeconomic determinants, for instance, changes in interest rate differential. The expression is modified in the following Eq. (2):

(2) $$\Delta {lnNER}_{t}=\Delta {OF}_{t}+\Delta W_{t}+\Delta {\left({IRSD}_{t}\right)+e}_{t}$$

Where $\Delta {lnNER}_{t}$ represent changes in the nominal exchange rates, $\Delta X_{t}$ is substituted with $\Delta {OF}_{t}$ , which represents daily order flow, the macroeconomic $\Delta I_{t}$ has been substituted with $\Delta \left({IRSD}_{t}\right)$ that represents domestic and foreign interest rates changes, and $e_{t}$ shows the error term.

The current study introduced an additional variable in the hybrid model, i.e., the bid-ask spread, that measures the liquidity of a currency [31]. found a significant explanatory power of order flow for active or highly traded currencies. They also highlighted that the bid-ask spread can explain the daily return in currencies with infrequent trade volume. Therefore, this study develops the extended macro-micro hybrid model, which includes cumulative order flow, bid-ask spread, and interest rate differential for long and short periods. The extended hybrid model (macro-micro determinants) is specified in Eq. (3):

(3) $$\Delta {lnNER}_{t}=\mu_{0}{+\mu_{1}\Delta {OF}_{t}+\mu_{2}\Delta lnBAS+\mu_{3}\Delta \left({IRSD}_{t}\right)+e}_{t}$$

where $\Delta {lnNER}_{t}$ represents the change in the exchange rate (MYR/USD), $\Delta {OF}_{t}$ represents the daily order flows, Δ *l*nBAS represents the bid-ask spread, $\Delta \left({IRSD}_{t}\right)$ represents the interest rate differential, $\mu^{\prime }s$ show the coefficients of variables, and $e_{t}$ denotes the error term.

Further, this study introduced an additional variable, oil price, as observable macro news to capture the combined influence of oil price and microstructure determinants. The model is supported by the studies of [16,39,40]. The extended model includes the oil price and the microstructure determinants of cumulative order flow and bid-ask spread. Equation (4) represent the extended hybrid model:

(4) $$\Delta {lnNER}_{t}=\mu_{0}{+\mu_{1}\Delta {OF}_{t}+\mu_{2}\Delta \ln\;{BAS}_{t}+\mu_{3}\Delta \left({lnOP}_{t}\right)+e}_{t}$$

Where *l*nOP represents the natural log of oil price. All the variables and parameters are already defined above.

There is growing literature on different topics such as psychology, politics, and economics, indicating that the individual's reaction is asymmetric to positive and negative information [26,59]. The prospect theory proposes a decision-making descriptive model that illustrates the individual's behavioral response more strongly to loss than gains. The theory was developed by Ref. [60]. Under the portfolio shift framework, order flow is signed as positive or negative, depending on whether the trade initiator is a buyer or seller of currencies [14]. The selling (buying) pressure indicates that foreign currency sales increase or decrease in favor of domestic currency. Given the growing nature of emerging markets like Malaysia, liquidity can be a critical factor in exchange rate determination. In the forex market, the spread is a commonly employed measure of liquidity. The bid-ask spread is important in determining the foreign exchange rate for low-trading currencies, implying that wider spreads indicate an increased risk of liquidity; however, narrower spreads represent less risk or more liquid markets.

Further, a rise in the interest rate differential leads to the depreciation of currency, as supported by Refs. [61,62]. Nevertheless, the sticky-price monetary model (SPMM) posits that an increasing trend in interest rate differential would attract foreign investment, which increases domestic currency demand and leads to currency appreciation [63]. In addition, the oil price acts as an observable macro-news predictor. Oil prices have significantly different impacts in oil-exporting and oil-importing countries. A rise or fall in oil prices signals good news or bad news for oil-exporting economies like Malaysia. The empirical studies provide evidence that oil prices responded more to negative shocks (bad news) than positive shocks (good news) in the short run [39,40]. Notably, low-traded exporting currencies provide a complex conclusion due to insensitive and illiquid markets. Hence, it is interesting to examine the influential determinants of asymmetric decision-making in Malaysia over a long and short period.

### 3.2 Empirical model

This paper used the extension of the autoregressive distributed lag (ARDL) approach as the nonlinear autoregressive distributed lag (NARDL) recommended by Ref. [64] Pesaran et al. (2001). The nonlinear ARDL lag specification originates from a 14 lag (p_max_ = q_max_ = 14), and all coefficients have been shown to be insignificant [[65], [66], [67]]. This paper decomposes the order flow into buying and selling pressures, the bid-ask spread into a wide and narrow spread, the interest rate differential into an increase or decrease in interest rate, and the oil price into macro news as good and bad news. Following [14], the hybrid model is augmented with bid-ask spreads and oil price predictors. The subsequent expression examines the impact of the hybrid approach of macro and micro determinants such as order flow, bid-ask spread, and interest rate differential on the exchange rate in the long and short periods for Model 1 is specified in Eq. (5):

(5) $$△{lnNER}_{t}=\alpha +\delta {lnNER}_{t-1}+\beta_{1}^{+}{OF}_{t-1}^{+}{+\beta }_{2}^{+}{OF}_{t-1}^{-}+\beta_{3}^{+}{lnBAS}_{t-1}^{+}{+\beta }_{4}^{+}{lnBAS}_{t-1}^{-}+\beta_{5}^{+}{IRSD}_{t-1}^{+}{+\beta }_{6}^{+}{IRSD}_{t-1}^{-}+\sum_{i=1}^{m}\lambda_{i}{△lnNER}_{t-i}+\sum_{i=0}^{m}({ⱷ}_{i}^{+}△{OF}_{t-i}^{+}+{ⱷ}_{i}^{-}△{OF}_{t-i}^{-})+\sum_{i=0}^{m}({ϐ}_{i}^{+}△{lnBAS}_{t-i}^{+}+{ϐ}_{i}^{-}△{lnBAS}_{t-i}^{-})+\sum_{i=0}^{m}(\varphi_{i}^{+}△{IRSD}_{t-i}^{+}+\varphi_{i}^{-}△{IRSD}_{t-i}^{-})+{\beta_{7}T}_{B}+\varepsilon_{t}$$

where $△{lnNER}_{t}$ represents the first difference operator of the nominal exchange rate, α is the intercept, $\sum_{i=0}^{m}\Delta$ shows partial sum changes, ${OF}^{+}$ and ${OF}^{-}$ represent the positive and negative order flow, respectively, ${lnBAS}^{+}$ and ${lnBAS}^{-}$ denote the positive and negative bid-ask spread, respectively, ${IRSD}^{+}$ and ${IRSD}^{-}$ denote the positive and negative interest rate differential, respectively, and $\varepsilon_{t}$ represents the white noise term.

Model 2 observes the exchange rate-hybrid approach of macro-micro determinants nonlinear nexus, such as cumulative order flow, bid-ask spread, and oil price in the long and short periods. Equation (6) is stated as follows:

(6) $$△{lnNER}_{t}=\alpha +\delta {lnNER}_{t-1}+\beta_{1}^{+}{OF}_{t-1}^{+}{+\beta }_{2}^{+}{OF}_{t-1}^{-}+\beta_{3}^{+}{lnBAS}_{t-1}^{+}{+\beta }_{4}^{+}{lnBAS}_{t-1}^{-}+\beta_{5}^{+}{lnOP}_{t-1}^{+}{+\beta }_{6}^{+}{lnOP}_{t-1}^{-}+\sum_{i=1}^{m}\lambda_{i}{△lnNER}_{t-i}++\sum_{i=0}^{m}({ⱷ}_{i}^{+}△{OF}_{t-i}^{+}+{ⱷ}_{i}^{-}△{OF}_{t-i}^{-})+\sum_{i=0}^{m}({ϐ}_{i}^{+}△{lnBAS}_{t-i}^{+}+{ϐ}_{i}^{-}△{lnBAS}_{t-i}^{-})+\sum_{i=0}^{m}({п}_{i}^{+}△{lnOP}_{t-i}^{+}+{п}_{i}^{-}△{lnOP}_{t-i}^{-})+\beta_{7}T_{B}+\varepsilon_{t}$$

where *ln*OP^+^ and *ln*OP^−^ show the positive and negative coefficients of oil price, respectively, and all the parameters have been defined above. Further, the asymmetric dynamic multipliers are used to show the association between unit changes in macro-micro determinants and nominal exchange rates. The cumulative effect of macro-micro determinants on the nominal exchange rate can be evaluated in four cases for Model 1 are explained from Eq. (7) to Eq. (10):

(7) $$△{lnNER}_{t}={\delta lnNER}_{t-1}+{Л}_{i}^{+}{{OF}_{t-1}^{+}}^{+}+{Л}_{i}^{-}{{OF}_{t-1}^{-}}^{+}+{ƛ}_{i}^{+}{{lnBAS}_{t-1}^{+}}^{+}+{ƛ}_{i}^{-}{{lnBAS}_{t-1}^{-}}^{+}+{п}_{i}^{+}{{IRSD}_{t-1}^{+}}^{+}+{п}_{i}^{-}{{IRSD}_{t-1}^{-}}^{+}+\sum_{i=1}^{p-1}\lambda_{i}{△lnNER}_{t-i}+\sum_{i=0}^{q-1}\left({⅄}_{i}^{+}△{OF}_{t-i}^{+}+{⅄}_{i}^{-}△{OF}_{t-i}^{-}\right)+\sum_{i=0}^{q-1}(o_{i}^{+}△{lnBAS}_{t-i}^{+}+o_{i}^{-}△{lnBAS}_{t-i}^{-})+\sum_{i=0}^{q-1}(\eta_{i}^{+}△{IRSD}_{t-i}^{+}+\eta_{i}^{-}△{IRSD}_{t-i}^{-})+e_{t}$$

(8) $$△{lnNER}_{t}={\delta lnNER}_{t-1}+{ЛOF}_{t-1}+{ƛlnBAS}_{t-1}+{пIRSD}_{t-1}+\sum_{i=1}^{p-1}\lambda_{i}{△lnNER}_{t-i}+\sum_{i=0}^{q-1}\left({⅄}_{i}^{+}△{OF}_{t-i}^{+}+{⅄}_{i}^{-}△{OF}_{t-i}^{-}\right)+\sum_{i=0}^{q-1}(o_{i}^{+}△{lnBAS}_{t-i}^{+}+o_{i}^{-}△{lnBAS}_{t-i}^{-})+\sum_{i=0}^{q-1}(\eta_{i}^{+}△{IRSD}_{t-i}^{+}+\eta_{i}^{-}△{IRSD}_{t-i}^{-})+e_{t}$$

(9) $$△{lnNER}_{t}={\delta lnNER}_{t-1}+{ЛOF}_{t-1}+{ƛlnBAS}_{t-1}+{пIRSD}_{t-1}+\sum_{i=1}^{p-1}\lambda_{i}{△lnNER}_{t-i}+\sum_{i=0}^{q-1}{⅄}_{i}{△OF}_{t-i}+\sum_{i=0}^{q-1}o_{i}{△BAS}_{t-i}+\sum_{i=0}^{q-1}\eta_{i}{△IRSD}_{t-i}+e_{t}$$

(10) $${lnNER}_{t}={\rho lnNER}_{t-1}+{\gamma OF}_{t-1}+{\vartheta lnBAS}_{t-1}+{oIRSD}_{t-1}+\sum_{i=1}^{p-1}\lambda_{i}{△lnNER}_{t-i}+\sum_{i=0}^{q-1}{⅄}_{i}{△OF}_{t-i}+\sum_{i=0}^{q-1}o_{i}{△BAS}_{t-i}+\sum_{i=0}^{q-1}\eta_{i}{△IRSD}_{t-i}+e_{t}$$

The expression of Model 2 examines the cumulative dynamic multiplier effects of the hybrid approach (order flow, bid-ask spread, and oil price) on the exchange rate for both the longer and shorter periods, as shown in Eq. (11) to Eq. (14) for Model 2:

(11) $$△{lnNER}_{t}={\delta lnNER}_{t-1}+{Л}_{i}^{+}{{OF}_{t-1}^{+}}^{+}+{Л}_{i}^{-}{{OF}_{t-1}^{-}}^{+}+{ƛ}_{i}^{+}{{lnBAS}_{t-1}^{+}}^{+}+{ƛ}_{i}^{-}{{lnBAS}_{t-1}^{-}}^{+}+\gamma_{i}^{+}{{lnOP}_{t-1}^{+}}^{+}+\gamma_{i}^{-}{{lnOP}_{t-1}^{-}}^{+}+\sum_{i=1}^{p-1}\lambda_{i}{△lnNER}_{t-i}+\sum_{i=0}^{q-1}\left({⅄}_{i}^{+}△{OF}_{t-i}^{+}+{⅄}_{i}^{-}△{OF}_{t-i}^{-}\right)+\sum_{i=0}^{q-1}(o_{i}^{+}△{lnBAS}_{t-i}^{+}+o_{i}^{-}△{lnBAS}_{t-i}^{-})+\sum_{i=0}^{q-1}({Ҩ}_{i}^{+}△{lnOP}_{t-i}^{+}+{Ҩ}_{i}^{-}△{lnOP}_{t-i}^{-})+e_{t}$$

(12) $$△{lnNER}_{t}={\delta lnNER}_{t-1}+{ЛOF}_{t-1}+{ƛlnBAS}_{t-1}+{\gamma lnOP}_{t-1}\sum_{i=1}^{p-1}\lambda_{i}{△lnNER}_{t-i}+\sum_{i=0}^{q-1}\left({⅄}_{i}^{+}△{OF}_{t-i}^{+}+{⅄}_{i}^{-}△{OF}_{t-i}^{-}\right)+\sum_{i=0}^{q-1}(o_{i}^{+}△{lnBAS}_{t-i}^{+}+o_{i}^{-}△{lnBAS}_{t-i}^{-})+\sum_{i=0}^{q-1}({Ҩ}_{i}^{+}△{lnOP}_{t-i}^{+}+{Ҩ}_{i}^{-}△{lnOP}_{t-i}^{-})+e_{t}$$

(13) $$△{lnNER}_{t}={\delta lnNER}_{t-1}+{Л}_{i}^{+}{{OF}_{t-1}^{+}}^{+}+{Л}_{i}^{-}{{OF}_{t-1}^{-}}^{+}+{ƛ}_{i}^{+}{{lnBAS}_{t-1}^{+}}^{+}+{ƛ}_{i}^{-}{{lnBAS}_{t-1}^{-}}^{+}+\gamma_{i}^{+}{{lnOP}_{t-1}^{+}}^{+}+\gamma_{i}^{-}{{lnOP}_{t-1}^{-}}^{+}+\sum_{i=1}^{p-1}\mu_{i}{△lnNER}_{t-i}+\sum_{i=0}^{q-1}{⅄}_{i}{△OF}_{t-i}+\sum_{i=0}^{q-1}o_{i}{△lnBAS}_{t-i}+\sum_{i=0}^{q-1}{Ҩ}_{i}{△lnOP}_{t-i}+e_{t}$$

(14) $${lnNER}_{t}={\delta lnNER}_{t-1}+{ЛOF}_{t-1}+{ƛlnBAS}_{t-1}+{\gamma lnOP}_{t-1}+\sum_{i=1}^{p-1}\mu_{i}{△lnNER}_{t-i}+\sum_{i=0}^{q-1}{⅄}_{i}{△OF}_{t-i}+\sum_{i=0}^{q-1}o_{i}{△lnBAS}_{t-i}+\sum_{i=0}^{q-1}{Ҩ}_{i}{△lnOP}_{t-i}+e_{t}$$

## 4 Results and discussion

Table 2 illustrates the summary of the descriptive data. The standard deviation of order flow indicates that the buying and selling of currencies are volatile at high frequency in the forex market. The result is supported by Ref. [68]. The oil price also confirms large variation due to the plunging in prices during the Global Financial Crisis from 2008 to 2010 and the commodity shock in 2014. Malaysia is a commodity exporter's economy that is affected by variations in global markets, particularly changes in the oil price [69]. Table 3 reports that the null hypothesis is rejected for univariate and multivariate series at a 5% level. The findings exhibit a nonlinear and random behavior of series, as supported by Ref. [70].

**Table 2:** Descriptive statistics.

Variables	Mean	Standard Deviation	Minimum	Maximum
*ln*NER	1.2475	0.1368	1.0774	1.5032
OF	0.3869	5.6072	−59.8230	70.7670
*ln*BAS	−5.6633	0.3947	−7.2644	−4.1997
IRSD	2.7197	0.3837	1.5800	3.1900
*ln*OP	4.3074	0.4006	3.1126	4.8254

**Table 3:** BDS statistics.

*M*	Univariate Series					Multivariate Series	
	*ln*NER	OF	*ln*BAS	IRSD	*ln*OP	Model-1	Model-2
2	0.2055	0.0809	0.0719	0.2015	0.2021	0.1143	0.1914
3	0.3486	0.1474	0.1315	0.3436	0.3438	0.2057	0.3250
4	0.4479	0.1944	0.1764	0.4431	0.4428	0.2695	0.4168
5	0.5167	0.2295	0.2059	0.5126	0.5115	0.3109	0.4790
6	0.5642	0.2516	0.2240	0.5617	0.5591	0.3363	0.5205

Note*:****m*** represents embedding dimension.

In the estimation process, the initial step is to confirm the order of integration. Table 4 illustrates that the observed variables show either an *I*(0) or an *I*(1) order of integration. Once unit root tests confirm the mixed integration process, this study proceeds to nonlinear ARDL (NARDL) estimation. Furthermore [71], tests indicate the time period for all the series. The nominal exchange rate time break is considered for further investigation to determine the exchange rate's behavior. The selected time period indicates the commodity market shock in 2014, in which the oil price plunge badly affected the Malaysian economy. Table 5 presents the Variance Inflation Factor (VIF) between the independent variables. VIF values exceeding 5 or 10 are a sign of significant multicollinearity, indicating a high correlation among the independent variables in the model. The result shows that all values are less than 5, which indicates no multicollinearity among independent variables.

**Table 4:** Unit root test results.

Variables	ADF		KPSS		ZA				Decision
	*I*(0)	*I*(1)	*I*(0)	*I*(1)	*I*(0)	Break Date	*I*(1)	Break Date	
*ln*NER	−0.3913	−43.5052*	4.7205*	0.2718	−4.3401	10/30/2014	−30.0577*	9/30/2015	*I*(1)
OF	−22.9639*	−15.9806*	0.1066*	0.0247*	−28.2743*	12/15/2015	−52.2863*	8/12/2015	*I*(0)
*ln*BAS	−3.1246**	−20.5027*	1.6218*	0.0784	−19.7059*	6/18/2015	−52.2829*	10/26/2015	*I*(0)
IRSD	−0.5932	−55.3890*	1.1103*	0.0630	−3.3963	7/01/2016	−37.1147*	5/03/2013	*I*(1)
*ln*OP	−1.0926	−35.8823*	3.5650*	0.1641	−4.0459	9/30/2014	−30.0685*	1/21/2016	*I*(1)

Note: *, **, and *** denote 1%, 5%, and 10% levels of significance, respectively.

**Table 5:** Variance inflation factor.

Variable	VIF
TB	1.30
LNBAS	1.27
IRSD	1.04
OF	1.00
LNOP	1.05
Mean VIF	1.132

**Fig. 2:**
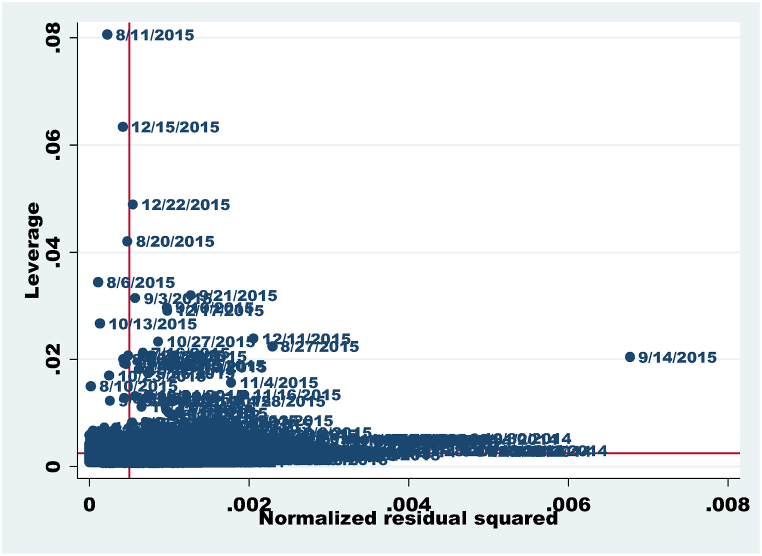
Normalized vs. Leverage Plot detecting outliers for model 1.

**Fig. 3:**
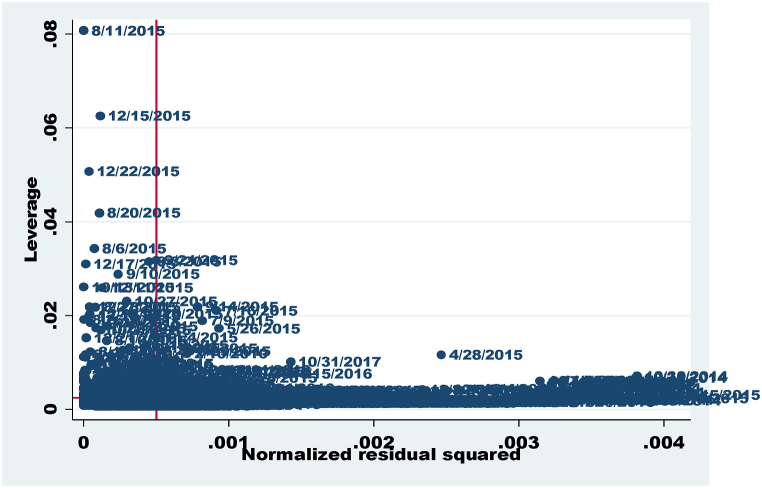
Normalized vs. Leverage Plot detecting outliers for model 2.

Moreover, Fig. 2, Fig. 3 display the Normalized vs. leverage Plot Detecting Outliers of both models. Both figures exhibit evidence of some outliers. There are some outliers in both figures. Outliers might frequently be present when dealing with daily data frequency in exchange rate determination. Robustness techniques can be used in the context of NARDL (Nonlinear Autoregressive Distributed Lag) analysis to deal with outliers and possibly reduce the impact of their influence on the estimation of parameters and standard errors. Thus, this study applied the robust standard errors in NARDL analysis. As it reduces the impact of outliers and other data features that violate the assumptions of conventional standard errors, it may result in more accurate and robust inference. The use of this is supported by Ref. [72].

Table 6 shows that the null hypothesis is rejected and implies the existence of a long-run cointegrating relationship between the exchange rate and the hybrid approach of macro-micro determinants in Malaysia. The findings are aligned with the studies of [16,40,73]. It suggests that commodity currencies are forward-leading indicators across commodity markets like Malaysia.

**Table 6:** Nonlinear ARDL bound test results for cointegration.

Bound test	Model-1	Model-2
F_PSS	9.7588	6.8868
99% Lower bound	5.17	5.17
99% Upper bound	6.36	6.36
Decision	Cointegration	Cointegration

Note: Critical values of F_PSS are taken with k = 3 for Model 1 and Model 2, respectively, at a 99% significance level.

Table 7 reports the long-run and short-run elasticities for both Models. A positive shock in order flow (OF) shows a positive effect on the exchange rate of about 0.014 and 0.013% in a single day for Model 1 and Model 2, respectively, at a 1% level in the long run. It implies that 1000 orders of dollars would decrease domestic currency demand, resulting in a decrease in the currency's value (appreciating the dollar). While a negative shock shows the negative linkage between order flow and exchange rate at a 1% significance level, suggesting appreciation of the currency by 0.014 for both models, A decrease in OF highlights the selling pressure on the dollar and increases the demand for Malaysian currency in the long run.

**Table 7:** Nonlinear ARDL estimates.

Variables	Model-1	Model-2
Constant	0.0141* (0.0042)	0.0124* (0.0040)
${\ln\;NER}_{t-1}$	−0.0133* (0.0036)	−0.0120* (0.0034)
${OF}_{t-1}^{+}$	0.0002* (0.0003)	0.0002* (0.0003)
${OF}_{t-1}^{-}$	0.0002* (0.0003)	0.0002* (0.0003)
$L_{{OF}_{t-1}}^{+}$	0.014* [0.001]	0.0131* [0.002]
$L_{{OF}_{t-1}}^{-}$	−0.014* [0.001]	−0.0135* [0.002]
${\ln\;BAS}_{t-1}^{+}$	0.0015* (0.0005)	0.0011** (0.0005)
${\ln\;BAS}_{t-1}^{-}$	0.0015* (0.0005)	0.0011** (0.0005)
$L_{\ln\;{BAS}_{t-1}}^{+}$	0.111** [0.018]	0.094** [0.043]
$L_{{\ln\;BAS}_{t-1}}^{-}$	−0.109* [0.018]	−0.095** [0.041]
${IRSD}_{t-1}^{+}$	−0.0011 (0.0010)	
${IRSD}_{t-1}^{-}$	−0.0010** (0.0005)	
$L_{{IRSD}_{t-1}}^{+}$	−0.086 [0.279]	
$L_{{IRSD}_{t-1}}^{-}$	0.073** [0.017]	
${\ln\;OP}_{t-1}^{+}$		−0.0011 (0.0008)
${\ln\;OP}_{t-1}^{-}$		−0.0021** (0.0010)
$L_{{\ln\;OP}_{t-1}}^{+}$		−0.093 [0.135]
$L_{{\ln\;OP}_{t-1}}^{-}$		0.178** [0.011]
${\Delta \ln\;NER}_{t-4}$	−0.0546** (0.0250)	−0.0535** (0.0242)
${\Delta \ln\;NER}_{t-12}$	0.0529** (0.0263)	0.0511** (0.0231)
$\Delta {OF}^{+}$	0.0001* (0.0003)	0.0001* (0.0003)
${\Delta OF}_{t-11}^{+}$		−0.0001*** (0.0001)
${\Delta OF}_{t-13}^{+}$		−0.0001*** (0.0004)
${\Delta OF}_{t-9}^{-}$	0.00001 (0.0002)	
${\Delta OF}_{t-12}^{-}$		0.0001*** (0.0004)
$\Delta {\ln\;BAS}^{+}$	−0.0015** (0.0007)	−0.0001** (0.0006)
${\Delta \ln\;BAS}_{t-2}^{+}$	−0.0014** (0.0007)	−0.0016** (0.0007)
${\Delta \ln\;BAS}_{t-12}^{+}$	−0.0009*** (0.0005)	
${\Delta \ln\;BAS}_{t-1}^{-}$	−0.0019** (0.0008)	−0.0016** (0.0007)
${\Delta \ln\;BAS}_{t-3}^{-}$	−0.0015** (0.0006)	−0.0016* (0.0006)
${\Delta \ln\;BAS}_{t-5}^{-}$	−0.0009*** (0.0005)	−0.0009** (0.0004)
${\Delta \ln\;BAS}_{t-6}^{-}$	−0.0016* (0.0005)	−0.0016* (0.0005)
${\Delta \ln\;BAS}_{t-7}^{-}$		−0.0008*** (0.0005)
${\Delta \ln\;BAS}_{t-9}^{-}$	−0.0011** (0.0005)	
${\Delta IRSD}_{t-11}^{+}$	0.0145*** (0.0078)	
${\Delta IRSD}^{-}$	−0.0059 (0.0051)	
$\Delta {\ln\;OP}^{+}$		−0.1023* (0.0160)
$\Delta {\ln\;OP}^{-}$		−0.1112* (0.0198)
T_B_	0.0019* (0.0006)	0.0003 (0.0007)
$W_{LR}OF$	1.012 [0.314]	3.052 [0.081]
$W_{SR}OF$	10.66 [0.001]	0.0559 [0.813]
$W_{LR}\;\ln\;BAS$	0.9396 [0.333]	1.01 [0.315]
$W_{SR}\;\ln\;BAS$	4.457 [0.035]	7.102 [0.008]
$W_{LR}IRSD$	0.0408 [0.840]	
$W_{SR}IRSD$	5.015 [0.025]	
$W_{LR}\;\ln\;OP$		8.972 [0.003]
$W_{SR}\;\ln\;OP$		0.0841 [0.772]

Note: *, **, and *** denote 1%, 5%, and 10% levels of significance, respectively. The values in () and [] represent standard error and p-value, respectively. The “+” and “-” show positive and negative sums, respectively. L^+^ and L^−^ denote the positive and negative changes of the long-run estimated coefficients, respectively. Δ refers to the short-run change, W denotes the Wald test, and LR and SR refer to the asymmetry condition in the long and short run, respectively.

Along with this, the result shows a positive correlation between order flow and exchange rate at a 1% level for both models in the short run. Relating to Model 2, the positive change in OF has a significantly adverse influence on the nominal exchange rate, denoting a selling pressure on the dollar and causing the appreciation of the Ringgit. Additionally, there is no significant impact of a negative change between these variables for Model 1. However, the negative change in OF highlights a positive effect on the exchange rate, implying that a 10% decrease in the OF specifies a depreciation of the exchange rate of about one basis point in the short run in Malaysia for Model 2. The results documented that OF shows a considerably different influence on the exchange rate in the short run for both models. Hence, the findings of this study infer that the oil price contains asymmetric information in the short run, which is helpful in the order flow information process to explain the exchange rate supported by Ref. [16].

The connection between order flow and exchange rate is similar to the study of [14], who found a strong correlation for highly traded currencies such as the Deutsche Mark/Dollar and Yen/Dollar at high-frequency data. Similarly [2], demonstrate a positive association between these variables in the Ugandan economy by using the ARDL framework over a long and short period. The results indicate that the market players in Malaysia are more active in dealing with currency buying and selling to improve the Ringgit price. The buying pressure of the US dollar is influential compared with the selling pressure in Malaysia. Furthermore, the findings confirm the asymmetry relation in the short run (W_SR_OF) for Model 1 and the long run (W_LR_OF) for Model 2.

Moreover, a positive shock in a bid-ask spread indicates significant results at a 5% level for both models over a long period. A rise in spread leads to upward movement in the exchange rate, reflecting the currency depreciation by 11% and 9% for Models 1 and 2, respectively. Furthermore, the negative shock in a bid-ask spread indicates the downward movement in the exchange rate for both models. The results imply that a decrease in the bid-ask spread would decrease the exchange rate and appreciate the domestic currency by 11% and 10% for Model 1 and Model 2, respectively, in the long run in Malaysia. The positive link between spread and exchange rate in the low-density trading market supports the concept of liquidity in the forex market. The findings conform to the past studies by Refs. [2,31] in the long run. The result is also supported by Ref. [74], who demonstrates that the Malaysian exchange rate is under speculative attack and that the Ringgit also experienced heavy selling pressure in the currency crisis. Both models' positive and negative shocks do not show any asymmetry for the longer time period in Malaysia.

The positive change in spread has negative effects on the exchange rate, with lag 2 at a 5% level for both models and lag 12 at a 10% level for Model 1. By contrast, the negative change in bid-ask spread coefficients has a negative influence on the exchange rate for both models. The results entail that a wider spread will appreciate the currency and a narrow spread will depreciate it, indicating speculative attacks on the Malaysian exchange rate in the short run. The results are in conformity with [29,75], who refer to the fact that spreads provide a premium return on low-trading countries [76]. demonstrate that a sharp increase in bid-ask spreads would increase record profits for banks. The results reveal the existence of short-run asymmetry for both models.

Besides, the long-run positive shock reports a negative but insignificant connection between the interest rate differential and the exchange rate in the long run, which is supported by Refs. [2,31], whereas a 5% decrease in the interest rate differential shows an appreciation of the exchange rate, which in turn decreases Malaysia's domestic currency over a long period. The results show a decrease in the interest rate discourages investment, thus increasing the foreign investment outflow and signaling a depreciation of the domestic currency. The result is in accordance with [63] overshooting hypothesis. In a shorter time, a positive change has a positive link with interest rate differential and exchange rate, stating that a rise in interest rate differential would depreciate the domestic currency, aligned with the uncovered interest rate parity theory. The result is in conformity with the past studies of [22,31], signifying a positive relationship, which was a common finding for the less developed economies in the shorter period. The convergence of negative shocks over a longer period is greater than that of positive shocks over a shorter period. Further, the findings confirm the asymmetry linkage between the interest rate differential and exchange rate in the short run (W_SR_IRSD) in Malaysia.

Table 7 also presents positive and negative changes in oil prices in the long and short runs in Malaysia under Model 2. The results report that a negative change has a negative linkage between the oil price and exchange rate at a 5% level in the long run. Moreover, a 5% decrease in oil prices would lead to the depreciation of the currency (upward movement of the exchange rate) by 18% over a longer period of time in Malaysia. Along with this, there is an inverse relationship between these variables for negative and positive change coefficients at a 1% level in a short period of time. It implies that a rise or fall in oil prices would appreciate or depreciate the currency by 10 and 11%, respectively. Further, negative shocks in oil prices have a stronger influence than positive shocks on the exchange rate in Malaysia. The results denote that the Malaysian market is responsive to variations in oil prices due to inefficiency in the Malaysian forex market.

The oil price acts as a macro-news announcement in a trade-oriented country like Malaysia. The results of this study show that traders seem to react more strongly to a decrease in oil prices. The findings are aligned with a theoretical phenomenon of portfolio balance introduced by Ref. [77], implying that an oil price decline will reduce the accumulated forex reserves through the wealth channel. The past studies of [[42], [43], [44]] discussed the association between oil prices and exchange rates. Apart from this, the changes in oil prices (positive and negative) show a significant difference in the asymmetric exchange rate over a longer period in Malaysia. Moreover, a long-run asymmetric relationship exists between these variables in Malaysia. However, the short-run asymmetric connection does not exist for a short period of time.

Moreover, the coefficient of the time break shows a significantly positive effect on the exchange rate at a 1% level for Model 1. The results suggest an unstable and uncertain situation in the Malaysian forex market that induced foreign investment outflows and speculative behavior in the currency market [78]. Finally, the error term exhibits the passive speed of adjustments in the presence of short-run market disequilibrium for both models. According to Ref. [65] less developed markets normally make adjustments slowly.

**Fig. 4:**
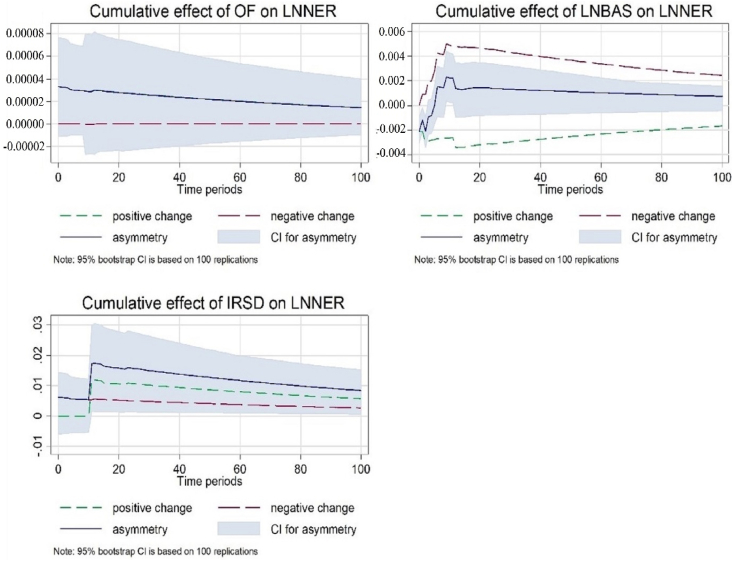
Dynamic multipliers capturing long-run symmetry and short-run asymmetry.

Fig. 4 shows the dynamic multipliers capturing long-run symmetry and short-run asymmetry. It exhibits the positive and negative lines (dashed green and red lines), which show the exchange rate adjustment caused by both positive and negative shocks. The deviation between positive and negative changes indicates an asymmetry curve in the blue line. Fig. 4 shows that a one percent shock in a negative change exhibits a positive connection between order flow (OF) and nominal exchange rate (NER), depicted by the red line. The decreasing trend of asymmetry is shown by a blue line, implying that a decrease in OF exerts dollar selling pressure and increases domestic currency demand. The findings are in accordance with the study of [65], who demonstrate that the selling pressure (negative OF) is stronger than that of the buying pressure (positive OF). Further, the graph outlines the cumulative asymmetry impact of spread on the exchange rate in the short period. Similarly, the decreasing change shows an opposite association between OF and NER. The result depicts the speculative attacks due to illiquid premiums in the forex currency market in Malaysia. It suggests that the bid-ask spread (BAS) causes an asymmetric impact on the exchange rate as liquidity costs increase in the economy, which is consistent with the studies of [29,75].

Moreover, all the short-run disequilibrium is corrected within 14 days. The BAS is the dominant determinant that has explanatory powers for frequently trading the currency of Malaysia in a short period of time. The result is supported by Ref. [31], who found greater explanatory power in bid-ask spread instead of order flow in low-trading currencies. Moreover, the graph of the cumulative effect of interest rate inferential (IRSD) on the exchange rate shows a strong asymmetry effect on the exchange rate in Malaysia for a short period. The one percent positive shock in IRSD presents a positive link between these variables.

**Fig. 5:**
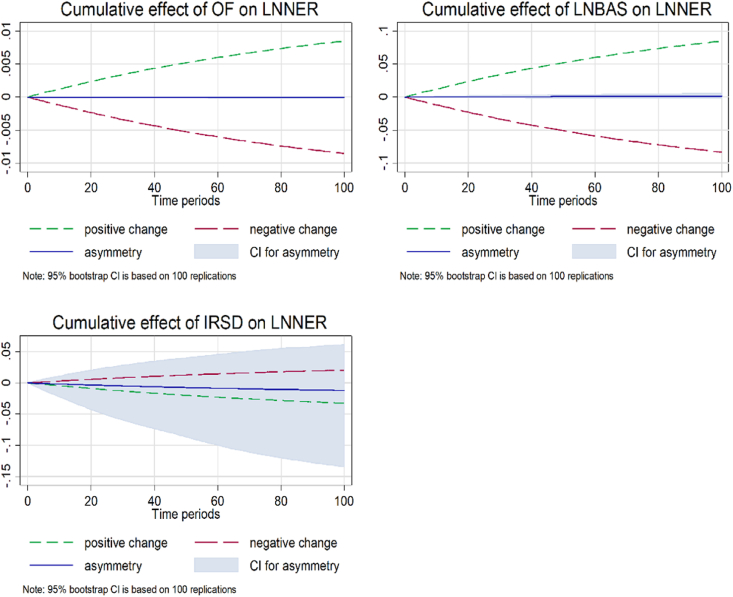
Dynamic multipliers capturing long-run asymmetry and short-run symmetry.

The sharp upward trend of asymmetry exhibits a rise in interest rates, which would lower domestic demand for currency and show an upward movement of the exchange rate, as supported by Ref. [22]. Fig. 5 depicts that the graph of and BAS shows no asymmetry effect, which is supported by Ref. [31], who confirm that microstructure determinants are the short-run factors. Additionally, there is the existence of a strong long-run asymmetric linkage between IRSD and NER. The IRSD and NER present an opposite nexus. A rise in IRSD leads to an appreciation of the currency, while a fall in IRSD shows a depreciation of the currency. The blue line indicates the increasing trend of asymmetry consistent with the UIP theory.

Fig. 5 exhibit the dynamic multipliers capturing long-run asymmetry and short-run symmetry. It displays an asymmetry graph of the cumulative effect of explanatory variables on the NER for Model 2 for the long and short periods. The graph of the cumulative effect exhibits a weak asymmetric association between microstructure determinants (OF and BAS) and the NER in the long and short runs in Malaysia.

**Fig. 6:**
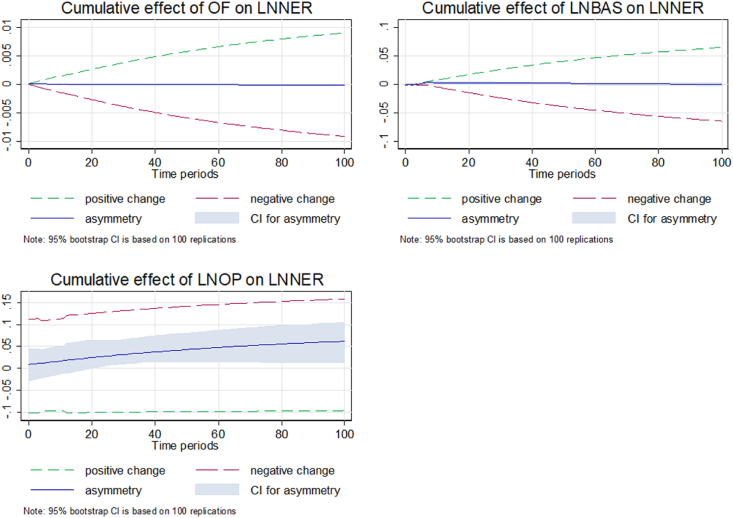
Dynamic multipliers capturing long-run and short-run asymmetry.

Fig. 6 present dynamic multipliers capturing long-run and short-run asymmetry. It displays the positive and negative shocks of the exchange rate and explains the buying and selling pressure of the Dollar (Ringgit) in Malaysia. The graph shows that the long- and short-period asymmetry is being corrected approximately within 14 days. OF expresses the dealer's positions in the forex market for a shorter period. Limited evidence shows the long- and short-period asymmetry adjustment patterns of the exchange rate [2,18]. Moreover, the graph shows weak evidence of an asymmetric effect between spread and NER in Malaysia. The findings of the asymmetry price discovery process in the presence of [31].

Additionally, dynamic multipliers capture the oil price (OP) and the NER asymmetry in the long and short periods. The graph shows a strong long-run and short-run asymmetric effect in Malaysia. The results exhibit a negative relationship between OP and NER. The decrease in OP positively affects the NER as it depreciates the domestic currency. However, increasing oil prices explains the negative effect on the NER, referring to the appreciation of the domestic currency. The results are in accordance with the theory, exhibiting that an oil price increase or decrease would appreciate or depreciate the export economy's currencies. The blue line shows the increasing trend of asymmetry with time. The asymmetry impact of the oil price can be examined by the past studies [79,80]. Hence, the result shows that oil price asymmetries appear important both in the long and short runs in Malaysia.

**Fig. 7:**
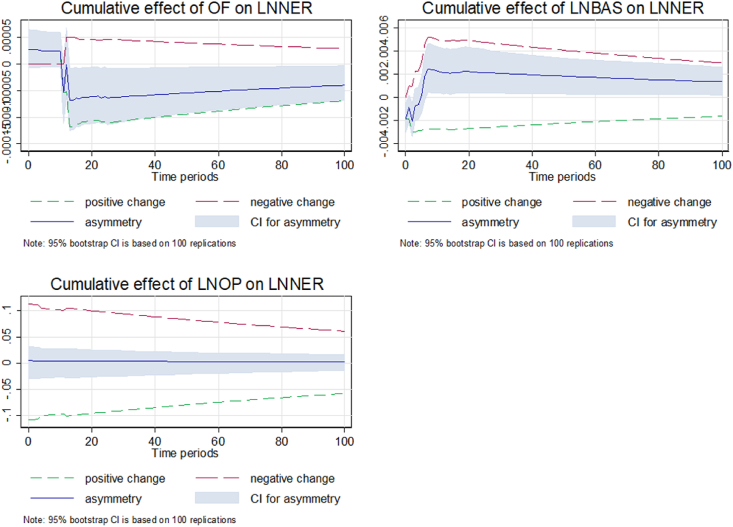
Dynamic multipliers capturing long-run symmetry and short-run asymmetry.

Fig. 7 shows dynamic multipliers capturing long-run symmetry and short-run asymmetry graphs of the cumulative effect of, *ln*BAS, and IRSD on the NER for Model 2. The positive (or negative) shock explains the buying and selling pressure of the Dollar (or ringgit) in Malaysia. Moreover, there is a weak correlation between BAS and NER related to the long-run and short-run asymmetric effects, consistent with [31]. Additionally, the results exhibit a negative OP-NER nexus, demonstrating that a decrease (increase) in OP has a positive (negative) influence on the NER as it depreciates (appreciates) the domestic currency. Hence, the findings support the evidence that an oil price increase or decrease would appreciate or depreciate the currencies of export economies like Malaysia.

**Fig. 8:**
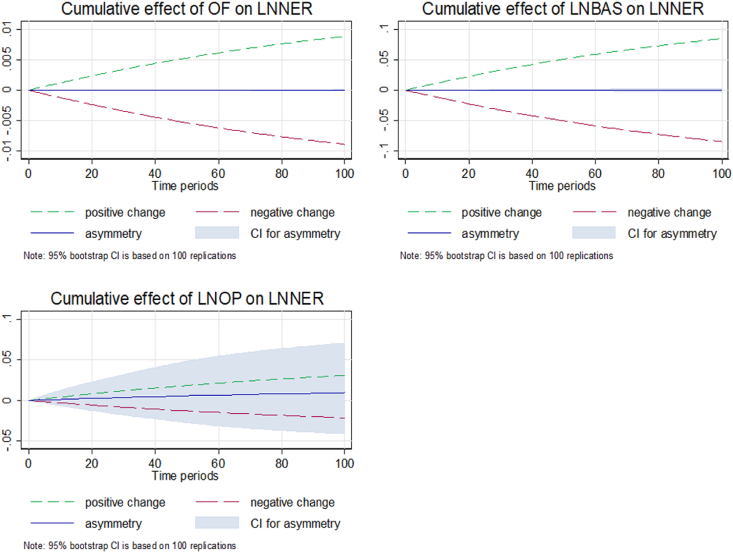
Dynamic multipliers capturing long-run asymmetry and short-run symmetry.

Fig. 8 presents the dynamic multipliers capturing long-run asymmetry and short-run symmetry of Model 2. The result is in line with Model 1, as the cumulative effect of and BAS does not provide evidence of asymmetry on the NER, consistent with the prominent study of microstructure determinants by Ref. [31]. In addition, the graph of the cumulative effect illustrates the long-run asymmetric effect between OP and NER in Malaysia. The results exhibit that an oil price plunge may deteriorate the forex reserves in an economy and eventually depreciate the exporting currency's value. However, increasing OP has a positive relationship with the NER. The long-run asymmetry price impact of OP is consistent with the theory and supported by the previous studies of [42,43,81].

The results of dynamic multipliers highlight that the OF and BAS provide strong evidence of the short-run asymmetries, while Malaysia provides a weak indication of the long-run asymmetry conditions for both models. Strong long-run and short-run asymmetry conditions exist between IRSD and NER for Model 1. In addition, OP as a news announcement indicator affects the NER in the long and short periods in Malaysia. The role of oil prices is influential in the longer period, but there is moderate proof of asymmetries in the shorter period of time in Malaysia.

Table 8 presents the diagnostic results showing that the inclusion of oil prices with microstructure determinants increased the variability of the overall model. It is noteworthy to mention that oil price as a macroeconomic news determinant accelerates the relationship in high-frequency data in Malaysia. The microstructure determinants indicate the prominent determinants of the exchange rate in the short run, while the role of interest rate differentials and oil prices as macroeconomic predictors has greater explanatory power in models. Table 8 also reports that there is no autocorrelation or heteroskedasticity issue in the data.

**Table 8:** Results of nonlinear ARDL diagnostic.

Model Diagnostic	Model-1	Model-2
R-squared	0.1292	0.2543
Adjusted R-squared	0.0805	0.2129
F-Stat	4.53 (0.0000)	10.53 (0.0000)
Serial Correlation^a^	44.72 (0.2802)	47.59 (0.1911)
Heteroskedasticity^b^	83.595 (0.0600)	74.752 (0.1110)

Notes: ^a^Portmanteau test statistics for serial correlation. *p*-value show in (). ^b^ Autoregressive conditional Heteroskedasticity (ARCH) test.

## 5 Conclusion

The findings of the current study provide several contributions to the existing body of knowledge related to nominal exchange rate determination over long- and short-run horizons. The outcome of the study produced an innovation drive in the growing body of nominal exchange rates and provided empirical evidence within emerging or low-traded currencies, particularly Malaysia. Thus, this study examined the microstructure approach to explain the exchange rate determination in long-run and short-run dynamics and included oil price announcements as macro-news announcements in the existing portfolio shift model. The findings of this study might be able to support the monetary policy makers in recognizing the nonlinearity involved in the microstructure approach that ought to be used in determining the exchange rate movements in Malaysia.

Thus, this paper aims to investigate the impact of a hybrid model of macro and micro determinants on the nominal exchange rate in the long and short runs. The sample period has been taken from January 4th, 2010 to December 29th, 2017 in the context of Malaysia. The results present a nonlinear connection between the exchange rate and the macro- and micro-determinants. This study also explores the fact that the response of forex traders is considerably asymmetrical to both positive and negative shocks. Based on this consideration, this study examines the market's asymmetric pricing impacts on the exchange rate by applying a dynamic asymmetric portfolio shift model and using the nonlinear ARDL approach.

We find greater magnitudes of the order flow and bid-ask spread for both models in the short run, whereas the interest rate differential and oil price have less magnitude in the shorter period than in the longer period for both models. The results also document that interest rate differentials and oil prices are influential predictors that explain the movement of exchange rate dynamics in the long run. As expected, the bid-ask spread emerged as the crucial factor that conveys the relevant information for the determination of the exchange rate. The study also finds that the pricing impact of the dollar-buying pressure would be more robust than the dollar-selling pressure, which eventually increases the liquidity cost. The preceding asymmetric price impacts support the evidence that traders are risk-takers in favorable markets. The result provides evidence that Malaysia's ill-liquid forex market provides the opportunity for speculative attacks.

Generally, the empirical findings of the hybrid approach supported the proposed dynamic asymmetric portfolio shift model. The results also highlighted that microstructure determinants and oil prices as “macro-news” are the influential determinants of the exchange rate for the Malaysian economy. It provides hidden information about the forex market. Moreover, the role of the oil price in microstructure determinants, particularly order flow, accelerates the price discovery process. Subsequently, the current study confirms that the bid-ask spread and oil price responded more strongly to negative shocks than positive shocks. Hence, it supports possible market asymmetric pricing impacts on the exchange rate due to oil prices and spread in the economy. Additionally, the Malaysian currency market is ill-liquid, which provides the opportunity for speculative attacks.

As a consequence, the results are significant for policymakers, academics, researchers, and financial institutions. The changes in the oil price and the nominal exchange rate linkage have received great concern from economists, investors, and policymakers in Malaysia. The results induced the Malaysian government to create a separate wealth fund to deal with the additional revenue received during the rise in oil prices. This policy is helpful in taking corrective measures as a consequence of the hike in oil prices. It is imperative to evolve suitable strategies to alleviate the harmful effects of oil prices on the Malaysian economy. Despite achieving significant objectives and contributions to research knowledge as well as to the research community, this study has limitations and some challenging areas for future research. The major limitation to exchange rate determination is the availability of microstructure data. There is a chance to carry out additional research into the factors that influence the exchange rate as more data becomes available. This would make it possible to compare the results of the current study and examine whether they are accurate or show any variations. In addition, another major limitation of the study is the availability of tick-by-tick currency order flow data, which was available from 2010 onward. Therefore, this study does not provide a comprehensive description of how currency order flow drives exchange rate movements before and after the Asian financial crisis and the global financial crisis.

Furthermore, recently, the COVID-19 pandemic had a greater impact on Malaysia's economy. Furthermore, a rapid and sustained decline in crude oil prices will naturally bring down oil-related revenue for the country. Thus, a critical evaluation of the combined impact of oil price and microstructure determinants on the exchange rate due to the COVID-19 pandemic offers an interesting research avenue. This paper employs a nonlinear ARDL approach to investigate the impact of a hybrid model of macro and micro determinants on the nominal exchange rate in the long run and short run. Extensions of our paper could employ other nonlinear approaches; see, for example [82], to investigate the impact of a hybrid model of macro and micro determinants on the nominal exchange rate in the long run and short run.

## Author contribution statement

Shamaila Butt: Analyzed and interpreted the data; Wrote the paper. </p>

Muhammad Ramzan: Conceived and designed the experiments; Performed the experiments; Supervision; Analyzed and interpreted the data. </p>

Wing-Keung Wong; Muhammad Ali Chohan; Suresh Ramakrishnan: Contributed reagents, materials, analysis tools or data. </p>

## Data availability statement

Data will be made available on request.

## Declaration of competing interest

The authors declare that they have no known competing financial interests or personal relationships that could have appeared to influence the work reported in this paper.
